# The Utility of *CYP2D6* and *CYP2C19* Variants to Guide Pharmacological Treatment in Complex Unipolar Major Depression: A Pilot Longitudinal Study

**DOI:** 10.1007/s11126-023-10044-9

**Published:** 2023-07-25

**Authors:** Reshma Ramaraj, Zeina N. Al-Mahayri, Reema Saleous, Karim Abdel Aziz, Fadwa Al-Mugaddam, Mouza Al-Sabousi, Aysha Alhassani, Noura Ali Al Ahbabi, Emmanuel Stip, George P. Patrinos, Bassam R. Ali, Danilo Arnone

**Affiliations:** 1 Department of Psychiatry and Behavioural Sciences, College of Medicine and Health Sciences, United Arab Emirates University, Al Ain, United Arab Emirates; 2 Department of Genetics and Genomics, College of Medicine and Health Sciences, United Arab Emirates University, Al Ain, United Arab Emirates; 3 Behavioural Science Institute, Al-Ain Hospital, United Arab Emirates Al Ain, Al-Ain, United Arab Emirates; 4 Université de Montréal, Institut Universitaire en Santé Mentale de Montréal, Montreal, Canada; 5 Department of Pharmacy, University of Patras School of Health Sciences, Patras, Greece; 6 Centre for Affective Disorders, Psychological Medicine, Institute of Psychiatry, King’s College London, London, UK; 7 Zayed Center for Health Sciences, United Arab Emirates University, Al Ain, United Arab Emirates

**Keywords:** Major depressive disorders, Unipolar major depression, *CYP2D6* and *CYP2C19* polymorphisms, Pharmacogenomics, Pharmacological treatment, Antidepressants, Treatment resistant depression

## Abstract

Major depression is a frequent condition which variably responds to treatment. In view of its high prevalence, the presence of treatment resistance in major depression significantly impacts on quality of life. Tailoring pharmacological treatment based on genetic polymorphisms is a current trend to personalizing pharmacological treatment in patients with major depressive disorders. Current guidelines for the use of genetic tests in major depression issued by the Clinical Pharmacogenomics Implementation Consortium (CPIC) are based on *CYP2D6* and *CYP2C19* polymorphisms which constitute the strongest evidence for pharmacogenomic guided treatment. There is evidence of increased clinical response to pharmacological treatment in major depression although largely in non-treatment resistant patients from Western countries. In this study, well characterised participants (N = 15) with complex, largely treatment resistant unipolar major depression were investigated, and clinical improvement was measured at baseline and at week-8 after the pharmacogenomics-guided treatment with the Montgomery Åsberg Depression Rating Scale (MÅDRS). Results suggested a statistically significant improvement (p = 0.01) of 16% at endpoint in the whole group and a larger effect in case of changes in medication regime (28%, p = 0.004). This small but appreciable effect can be understood in the context of the level of treatment resistance in the group. To our knowledge, this is the first study from the Middle East demonstrating the feasibility of this approach in the treatment of complex major depressive disorders.

**Supplementary Information:**

The online version contains supplementary material available at 10.1007/s11126-023-10044-9.

## Introduction

Depressive disorders are frequently occurring conditions globally with a high prevalence, a tendency to chronicity, and variable response to treatment [1]. The tendency to treatment refractoriness in unipolar major depression increases with the number of pharmacological trials [2]. According to the Sequenced Treatment Alternatives to Relieve Depression (STAR*D) study, evaluating treatment response in major depression across four standardised progressive levels of treatment, the theoretical cumulative remission rate of 67% is more likely to occur at the first two treatment stages (20–30%), rather than the subsequent ones (10–20%) [3, 4]. Overall, 10–30% of cases of unipolar depression become resistant to treatment, of which 30% display residual symptoms, treatment unresponsiveness, and impaired level of social and occupational functioning. These symptoms are often aggravated by suicidal ideation and a decline in physical health [5]. It is therefore important to consider approaches with the potential for ameliorating clinical outcomes at the earliest possible stage of illness.

A current approach to personalized medicine includes the use of pharmacogenomics to guide pharmacological management by taking into consideration individual genetic variabilities [6]. In a recently published meta-analysis of randomized controlled trials, we demonstrated that pharmacogenomic testing which include *CYP2D6* and *CYP2C19* genetic variants is a useful tool to increase effectiveness of antidepressant treatment in major depressive disorders with odds for improvement, response, and remission in the range of 1.46–1.85 compared to treatment as usual [7]. Current guidelines for the use of genetic tests in major depression issued by the Clinical Pharmacogenomics Implementation Consortium (CPIC; www.cpicpgx.org) are based on variants in these two genes which constitute the strongest evidence for pharmacogenomic guided treatment in major depression [8, 9]. It is noteworthy that the studies that tested the impact of pharmacogenomics in major depressive disorders and included *CYP2D6*/*CYP2C19* genetic variants largely investigated participants at the beginning of their treatment histories where the impact of pharmacogenomics could be more significant due to the prevailing absence of treatment-resistant depression [7]. This is in line with a recent study by Fan and Bousman which estimated that up to one-third of US and Canadian patients treated for major depressive disorders carry actionable *CYP2C19* and *CYP2D6* genetic variants and could benefit clinically from pairing *CYP2C19* and *CYP2D6* testing with the STAR*D treatment algorithm conferring greater effect of *CYP2C19* genotyping for the first two steps and *CYP2D6* genotyping for the remaining 3 steps [10]. Although Fan and Bousman’s results are consistent with a cumulative probability estimate for the frequency of non-normal metaboliser phenotypes of *CYP2D6* and *CYP2C19* across different populations (36.4% for *CYP2D6* and 61.9% for *CYP2C19*) [11], the impact of this approach in the Middle East is largely unknown due to the absence of systematic research conducted in this part of the world [11].

The work presented here is a feasibility study which evaluated the impact of pharmacogenomics in treating complex presentations of unipolar major depression in the Middle East, where to our knowledge, no previous similar studies have been carried out. The study was conducted in a tertiary centre for mood disorders where higher levels of treatment resistance prevail. Herein, we hypothesized that pharmacogenomics would contribute to improve clinical outcomes, although we expected an overall lower efficacy than in clinical settings where treatment-resistant patients are excluded.

## Methods

### Inclusion and Baseline Assessments

Subjects were assessed in the context of a tertiary-level mood disorder clinic based at Al-Ain Hospital, Abu Dhabi, United Arab Emirates. Patients were referred to the clinic by secondary care clinicians for an evaluation and treatment plan formulation. An essential criterion for referrals was experiencing an episode of unipolar major depression. Participants received a baseline assessment and were offered 4-week interval follow-ups for up to three months. Major depression was assessed and diagnosed according to the Mini International Neuropsychiatry Interview for DSM-IV [12]. Each patient also received ICD-10 diagnostic codes in agreement with local requirements for recording clinical diagnoses in the electronic record system [13]. Psychiatric comorbidities were not an exclusion criterion. Written informed consent was obtained from all subjects involved in the study before their enrolment. The study was conducted according to the guidelines of the Declaration of Helsinki and approved by the United Arab Emirates University Human Research Ethics Committee (ERH-2020-6134 2020-09) on 17/09/2020 with an extension on 03/08/2022, the Al Ain hospital Research Ethics Governance Committee (Ref.: AAHEC − 05-20-014) on 11/06/2020 and the Abu Dhabi Health Research and Technology Ethics Committee (Ref.: DOH/CVDC/2022/1107) on 17/05/2022.

At baseline, clinical characterization included an assessment of the level of treatment refractoriness by using the Maudsley staging method (MSM). The MSM provides a comprehensive standardized graded assessment of the level of treatment resistance in unipolar major depression based on the severity of symptoms, course of illness, and failed treatments/use of augmentation/electroconvulsive therapy (ECT) [14]. In addition, baseline severity was assessed with the Clinical Global Impression-Severity (CGI-S) scale [15], the Montgomery Åsberg Depression Rating Scale (MÅDRS) for depressive symptoms [16], and Hamilton rating scale for anxiety symptoms (HAM-A) [17]. Symptoms of elation were excluded with the Young Mania rating scale (YMRS) [18], and level of functioning was assessed with the Global Assessment of Functioning (GAF) [19]. Bipolar diathesis was excluded by the Mood Disorder Questionnaire (MDQ) [20].

### Pharmacogenomic Testing

Participants were offered a pharmacogenomic test evaluating the common actionable alleles of *CYP2D6* and *CYP2C19* at baseline. The test included genotyping of the single nucleotide polymorphisms (SNPs) determining the common actionable alleles in both genes. Taqman® SNP Genotyping Assays and Taqman® genotyping master-mix (Applied Biosystems, ThermoFisher Scientific) were used to detect CYP2C19*2/*3/*6/*9/*17 and CYP2D6*3/*4/*6/*9/*10/*40/*41 alleles. The details of tested variants are listed in Supplementary Table S1. The copy number variation of *CYP2D6* (i.e., whole gene deletion (CYP2D6*5) or duplication) was assessed through long-range PCR as described earlier [21]. The absence of any alternative alleles at the examined SNPs and copy number variation was considered as carrying the *1/*1 alleles.

The pharmacogenomic results were translated then into clinical recommendations depending on the latest CPIC recommendations [8, 9]. The reports with pharmacogenomic results and clinical recommendations were provided to their treating clinicians within seven days after recruitment to be acted upon.

### Primary Outcome

Changes in MÅDRS rating scale scores from baseline to week-8 were used as a primary outcome measure for improvement (reduction in rating scale score), response (≥50% MÅDRS reduction), and remission (< 7 MÅDRS score). DA and RA administered the MÅDRS, and the inter-rater reliability correlation coefficient calculated between assessors was 0.87.

### Statistical Analysis

Demographic data were summarized with descriptive statistics (means and standard deviations, SD). The normality of the sample distribution was assessed with the Shapiro-Wilk test. Patients’ rating scale scores mean difference at baseline and week 8 were compared using a paired two-sample t-test. In case of missing values, the most conservative method of the last observation available at week-4 was carried forward to week-8. The level of statistical significance was set at p ≤ 0.05, two tail distribution. Statistical tests were applied using SPSS (IBM) version 28.

## Results

In the period between January 2022 and December 2022, 17 patients were identified as suitable candidates, met the study inclusion criteria and 16 consented to participate. One participant was excluded at study entry due to abnormal liver function impacting on cytochrome P450 activity. Fifteen currently depressed patients were included in the study and their clinical and demographic characteristics are illustrated in Table 1. All met the criteria for a depressive episode (F32) or recurrent depressive disorder (F33). Nine patients experienced co-morbidities which included social anxiety, specific phobia, generalized anxiety disorder, panic disorder, post-traumatic stress disorder, misuse of alcohol, obsessive-compulsive disorder, and attention deficit hyperactivity disorder. None experienced any medical condition which interfered with study analysis or interpretation.

**Table 1:** Clinical and demographic characteristics of the participants. Course of the current episode according to the Maudsley Staging Method, A: acute (≤12 months), SA: subacute (13–24 months), C: Chronic (> 24 months). AD: antidepressant. UAE: United Arab Emirates

Patient ID	Course	N. Failed pharmacological trials	ECT	Augmentation	Previous episodes	Age	Sex	Nationality	AD regime modification
PS1	A	1	No	No	0	56	Female	India	AD dose increase
PS2	C	2	No	No	3	37	Female	Egypt	AD combination
PS3	C	4	No	Yes	0	35	Male	UAE	AD combination
PS4	C	4	Yes	Yes	0	30	Male	UAE	AD discontinuation
PS5	A	1	No	No	0	52	Male	India	No change
PS6	A	2	No	Yes	0	23	Male	Syria	AD change
PS7	C	1	No	Yes	0	28	Female	Pakistan	AD combination
PS8	SA	2	No	Yes	0	22	Male	Pakistan	No change
PS9	C	4	No	Yes	0	44	Female	UAE	No change
PS10	SA	5	Yes	Yes	3	40	Female	UAE	AD combination
PS11	C	4	No	Yes	2	27	Male	UAE	AD change
PS12	C	3	No	Yes	2	27	Male	UAE	AD combination
PS13	C	5	No	No	2	34	Male	UAE	No change
PS14	SA	1	No	No	2	31	Female	Somalia	No change
PS15	C	4	Yes	Yes	4	58	Male	Afghanistan	No change

The participants’ mean age was 36 years, 6 women and 9 men. Seven participants were from the United Arab Emirates, two from India, two from Pakistan, and one from Egypt, Syria, Afghanistan, and Somalia. Patients’ characteristics at study entry included a mean MÅDRS score of 21.53 (SD: 8.62) in the moderate range, CGI severity baseline score of 3.87 (SD: 0.99) also indicating an average moderate level of severity, a GAF mean score of 58.67 (SD: 7.43) suggesting a significant impact on level of functioning. The mean MSM score was 8.8 (SD: 2.91) suggesting an average moderate level of treatment resistance. At the time of enrolment, the HAM-A score was 10.02 (SD: 6.87) suggesting mild anxiety levels. YMRS mean score of 1.73 suggested no evidence of elation (SD: 1.79). There was no evidence of bipolar diathesis according to the MDQ.

Pharmacogenomic testing results, including the *CYP2C19* and *CYP2D6* alleles, the predicted metabolic status, and the resulting clinical recommendations depending on CPIC guidelines are illustrated in Table 2. In summary, only five patients (33%) carried wild-type alleles for both *CYP2D6* and *CYP2C19* genes with a predicted normal metabolic activity. In comparison, 2 (13%), 3 (20%), and 4 (27%) patients were rapid, poor, and intermediate *CYP2C19* metabolizers, respectively, while one patient (6%) and 3 patients (20%) were *CYP2D6* poor and intermediate metabolizers, respectively.

**Table 2:** Patients’ pharmacogenomic results and the resulting clinical recommendations for doses of anti-depressants according to the CPIC guidelines [8, 9]. *The ✓ symbol designates where the medication can be initiated with the standard starting dose

Patient ID	*CYP2C19* alleles [Metabolic status]	*CYP2D6* alleles [Metabolic status]	Citalopram/ Escitalopram	Sertraline	Paroxetine	Fluvoxamine	Nortryptiline/ Desipramine	Amitriptyline
1	*2/*2 [Poor metabolizer]	*1/*1 [Normal metabolizer]	Consider 50% reduction of starting dose	Consider 50% reduction of starting dose	✓	✓	✓	Avoid
2	*1/*1 [Normal metabolizer]	*1/*10 [Normal metabolizer]	✓	✓	✓	✓	✓	✓
3	*1/*1 [Normal metabolizer]	*1/*1 [Normal metabolizer]	✓	✓	✓	✓	✓	✓
4	*1/*17 [Rapid metabolizer]	*10/*40 [Intermediate metabolizer]	Consider an alternative	✓	✓	✓	Consider 25% reduction of starting dose	Consider an alternative
5	*2/*2 [Poor metabolizer]	*1/*10 [Normal metabolizer]	Consider 50% reduction of starting dose	Consider 50% reduction of starting dose	✓	✓	✓	Avoid
6	*1/*1 [Normal metabolizer]	*2/*2 [Normal metabolizer]	✓	✓	✓	✓	✓	✓
7	*1/*2 [Intermediate metabolizer]	*1/*2 [Normal metabolizer]	✓	✓	✓	✓	✓	✓
8	*2/*2 [Poor metabolizer]	*2/*41 [Normal metabolizer]	Consider 50% reduction of starting dose	Consider 50% reduction of starting dose	✓	✓	✓	Avoid
9	*1/*2 [Intermediate metabolizer]	*1/*10 [Normal metabolizer]	✓	✓	✓	✓	✓	✓
10	*2/*17 [Intermediate metabolizer]	*1/*1 [Normal metabolizer]	✓	✓	✓	✓	✓	✓
11	*1/*1 [Normal metabolizer]	*2/*2 [Normal metabolizer]	✓	✓	✓	✓	✓	✓
12	*1/*1 [Normal metabolizer]	*1/*2 [Normal metabolizer]	✓	✓	✓	✓	✓	✓
13	*1/*17 [Rapid metabolizer]	*41/*41 [Intermediate metabolizer]	Consider an alternative	✓	✓	✓	Consider 25% reduction of starting dose	Consider alternative
14	*1/*1 [Normal metabolizer]	*5/*5 [Poor metabolizer]	✓	✓	Select an alternative	Consider 25–50% reduction of starting dose	Avoid	Avoid
15	*2/*17 [Intermediate metabolizer]	*1/*40 [Intermediate metabolizer]	✓	✓	✓	✓	Consider 25% reduction of starting dose	Consider 25% reduction of starting dose

The pharmacogenomic-based reports including the resulting recommendation were shared with the treating clinicians who considered these recommendations with the patient’s clinical presentation, concomitant medications, and history of antidepressants use. The pharmacogenomic-based recommendations were taken into account when modifying the pharmacological management in 60% of cases (N = 9) during the study period and included antidepressant type/class change, augmentation, and dose change (Table 1).

**Fig. 1:**
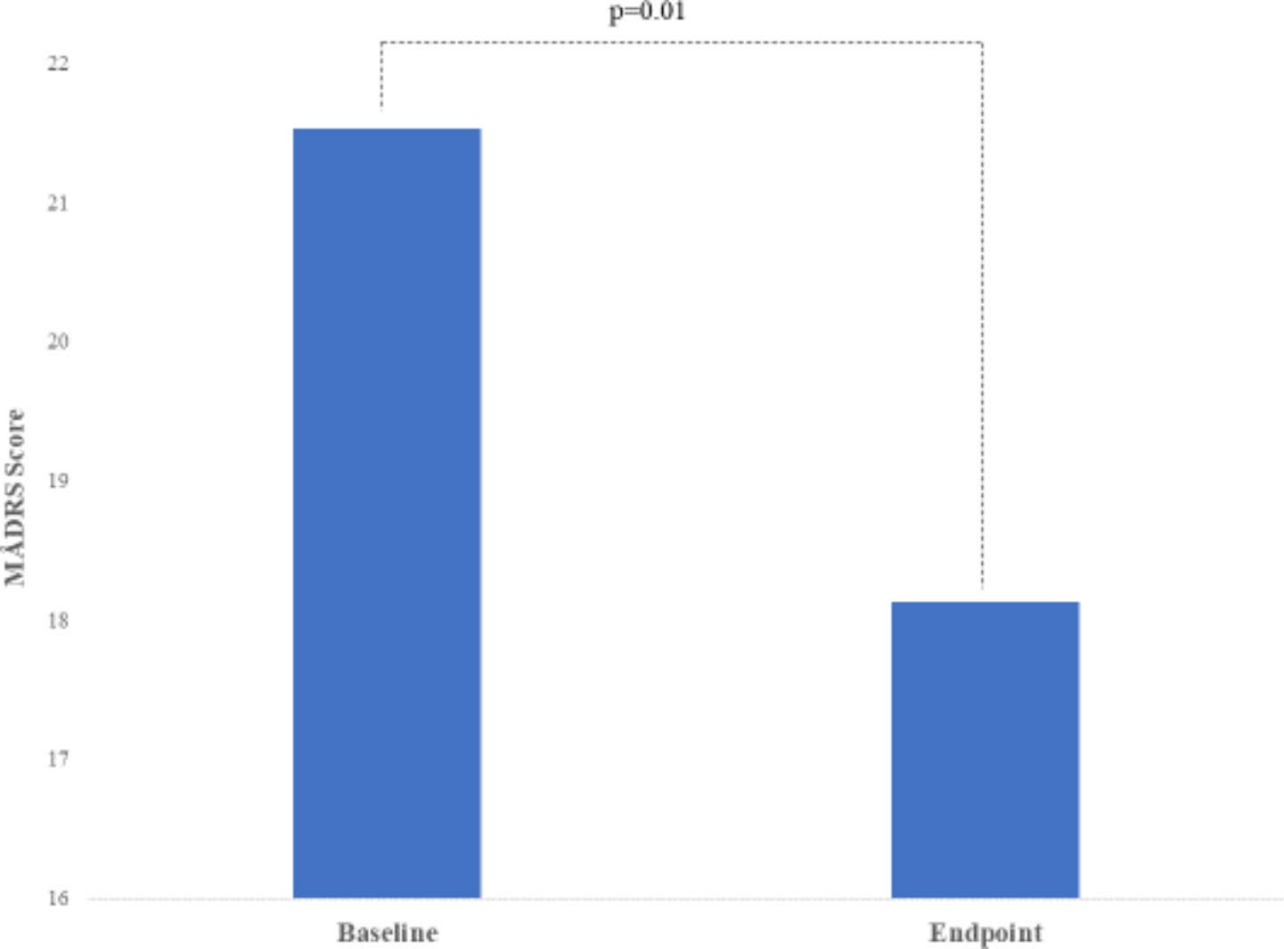
Differences in MÅDRS depression rating scale scores from baseline to endpoint (week 8) for the whole group (N = 15)

Statistical analysis to measure the change in the primary outcome suggested a 16% reduction in mean MÅDRS scores at week-8 from 21.53 (SD: 8.62) to 18.13 (SD: 8.61) for the whole group of 15 participants. Although improvement was widespread and variable, none of the patients achieved ≥50% reduction on the MÅDRS compatible with response, and only one patient remitted (< 7 MÅDRS score). Figure 1 shows the result of the paired t-test (N = 15) suggesting that the difference in MÅDRS scores was statistically significant (p = 0.01). The sub-analysis of the 9 participants whose medication regime was modified indicated a larger change in MÅDRS score of 28% which was statistically significant (p = 0.004).

## Discussion

In this work, we evaluated the impact of a pharmacogenomic-guided approach on clinical improvement to treat unipolar major depression. The results indicate a measurable reduction in MÅDRS scores in the range of 16% in a group of depressed patients with overall complex clinical presentations who received pharmacogenomic testing over a period of 8 weeks. Our results are consistent with our recent meta-analysis which reported that the odds for improvement with a pharmacogenomic approach which included *CYP2D6* and *CYP2C19* genotypes versus treatment as usual are increased by 63% [7]. Current guidelines for the use of genetic tests in major depression issued by the CPIC are based on variants in these two genes which constitute the strongest evidence for pharmacogenomic guided treatment in major depression [8, 9].

Although the percentage of improvement in this study is small in magnitude, the impact on individuals with overall significant levels of treatment refractoriness, chronic symptoms, and impaired level of function is not to be underestimated. In treatment-resistant depression, those who remain in the episode after treatment failures are characterized by a poor longitudinal outcome [22]. Hence, small therapeutic gains in this group can have beneficial effects in relation to symptomatic relief and quality of life.

Although several studies have evaluated the effect of pharmacogenomic-guided depression treatment in treatment-naïve patients [7], to our knowledge, only McCarthy and colleagues conducted a randomized controlled trial in treatment-resistant depression [23]. The authors reported that although remission rates in the pharmacogenomic guided group were higher than the treatment as usual group measured at endpoint with CGI scores (29% vs. 21%), the difference between the two groups did not reach statistical significance. Our findings are largely in agreement with McCarthy and colleagues’ and support an association between the use of pharmacogenomics and clinical improvement measured with MÅDRS. However, the absence of a control group, the lack of randomisation and the small number of participants, prevents any definitive conclusion about the nature of the association and cannot exclude the possibility that improvement could have supervened independently from the use of pharmacogenomics. Other potentially relevant differences between the two studies include the type of participants. In McCarthy and colleagues’ work patients were veterans recruited from a ‘real world’ clinical environment, in our study the majority of patients were recruited from a tertiary level mood disorder service with a mean MSM score of 8.8. McCarthy and colleagues also evaluated the responses of the clinicians in relation to the use of the test to guide pharmacotherapy which was perceived useful in 57% of cases. The authors reported that amelioration of side effects was the primary indication for the use of the test by clinicians, followed by a reduction in side effects and increased efficacy, and improved efficacy in a minority of cases. Interestingly clinicians in McCarthy and colleagues’ study reported that the test was not particularly useful in dosing [23]. We did not formally collect information from clinicians systematically and it is therefore not possible to reliably comment on clinicians’ feedback from this study.

It is noteworthy that CPIC evidence-based recommendations are primarily centred on selective serotonin reuptake inhibitors (SSRIs) and tricyclics antidepressants [8, 9]. SSRIs are commonly used as first line pharmacological treatment in major depressive disorders [1]. This might contribute to explain the larger impact of pharmacogenomic guided treatment in major depression for depressed patients who are relatively new to pharmacological treatment [10] and the large effect size supporting pharmacogenomic guided treatment shown in meta-analyses which largely excluded treatment resistance [7]. In addition, with an increasing number of treatment trials, improvement, response and remission rates significantly diminish as shown in the STAR*D study [3], contributing to explain the limited level of improvement in this work. An additional possibility is that in the Middle East the frequency of non-normal metaboliser phenotypes of *CYP2C19* and *CYP2D6* might be inferior to other ethnic groups around the world, reducing the overall spectrum of actionable genotypes for pharmacogenomic guided treatment in major depression.

It is of interest that 66% of the tested patients had at least one impaired function allele at *CYP2C19* and *CYP2D6*. This observation is consistent with previous reports that almost 70–80% of individuals affected by mental health conditions, carry at least one impaired allele at *CYP2C19* and/or *CYP2D6* [24]. Indeed, both genes show high interethnic variability. The patients in the current cohort were primarily from the Middle East and South Asia, and the detected frequencies of *CYP2C19* poor and ultrarapid alleles are within the frequencies of the same alleles in Middle Eastern and South Asian subgroups [25]. Similarly, the reported low frequency of *CYP2D6* poor function alleles is a common observation in Middle Eastern populations [26].

Further limitations of this study include the small sample size reflecting the exploratory nature of the work designed to evaluate the feasibility of this type of research in the Middle East and estimate the effect size necessary to confer sufficient statistical power. Research in mood disorders presents significant challenges, and pilot studies can provide essential guidance to help define the number of participants to sufficiently power research work [27]. The effect size from this study, calculated according to the formula d = μ1 − μ2/σ (where μ1 and μ2 are the mean MÅDRS scores for the two visits and σ is the standard deviation of one of the two time points) is equal to 0.39, considered in the moderate range [28]. Based on this calculation, the a-priori number of participants necessary to power a study to evaluate the effect of pharmacogenomics vs. treatment as usual with a two tailed distribution, a probability for error of α = 0.05, a power of 1- β = 0.8 and a 1:1 allocation ratio is 172 participants in each group [29]. Recruiting an actual sample of this size (N = 344) in the Middle East, especially in view of the likely significant attrition rate for longitudinal studies, is likely to be challenging and require a broad collaboration across several sites and centres in the United Arab Emirates (UAE) and beyond in the Middle East to create a shared protocol and a central database. This promising project could provide very informative results in view of the multi ethnicity of this region and the scarcity of similar research originating from this part of the world. In addition, the use of pharmacogenomics in the treatment of common psychiatric disorders such as major depression could be a useful cost saving strategy which could have a wider impact in less affluent countries in the Middle East and North Africa that share a similar ethnic distribution [30].

## Conclusion

This study provides evidence supporting the feasibility of introducing pharmacogenomic-guided treatment in psychiatric clinics in the UAE. To our knowledge, the current study is the first attempt in the country and the region. Our data show that antidepressant treatment guided by pharmacogenomic testing of *CYP2D6* and *CYP2C19* could add incremental benefits to clinical response even in the presence of challenging treatment-resistant presentations. Genotyping for *CYP2D6* and *CYP2C19* variants in the context of specialized mood disorder services that offer tailored interventions to complex patients is suggested here as an additional supportive approach to complement available innovative technologies [31, 32].

## Electronic Supplementary Material

Below is the link to the electronic supplementary material.

Supplementary Material 1

## Data Availability

The data presented in this study are available upon request from the corresponding author. The data are not publicly available due to privacy restrictions.
