# Supplementary material for: The Utility of CYP2D6 and CYP2C19 Variants to Guide Pharmacological Treatment in Complex Unipolar Major Depression: A Pilot Longitudinal Study
Source: Psychiatr Q. 2023 Jul 25;94(3):435–47. doi: 10.1007/s11126-023-10044-9 (PMC10460303; doi:10.1007/s11126-023-10044-9)
Supplement: Supplementary file 1 — Supplementary Material 1 [file 11126_2023_10044_MOESM1_ESM.docx]

***CYP2D6* and *CYP2C19* variants to guide pharmacological treatment in complex unipolar major depression: a pilot longitudinal study.**

**Supplementary Material**

**Table S1: Tested variants on *CYP2C19* and *CYP2D6* and their associated alleles**

| Gene | Variant rs-number | Star allele^1^ | Ref | Alt |
| --- | --- | --- | --- | --- |
| CYP2C19 | rs4244285 | *2 | G | A |
| CYP2C19 | rs12769205 | *2/*35 | A | G |
| *CYP2C19* | rs4986893 | *3 | G | A |
| *CYP2C19* | rs12248560 | *17 | C | T |
| *CYP2C19* | rs28399504 | *4 | A | G |
| *CYP2D6* | rs35742686 | *3 | T | del T |
| *CYP2D6* | rs3892097 | *4 | C | T |
| *CYP2D6* | rs5030655 | *6 | A | del A |
| *CYP2D6* | rs5030656 | *9 | TCT | del TCT |
| *CYP2D6* | rs1135840 | *2/*10/*40/*41 | C | G |
| *CYP2D6* | rs1065852 | *10 | G | A |
| *CYP2D6* | rs16947 | *2/*40/*41 | G | A |
| *CYP2D6* | rs28371706 | *40 | G | A |
| *CYP2D6* | rs28371725 | *41 | C | T |

**^1^ Star alleles defined by their core SNPs as designated in** [**www.pharmvar.org**](http://www.pharmvar.org)
